# Guidance for otolaryngology health care workers performing aerosol generating medical procedures during the COVID-19 pandemic

**DOI:** 10.1186/s40463-020-00429-2

**Published:** 2020-06-03

**Authors:** Marc J. W. Lammers, Jane Lea, Brian D. Westerberg

**Affiliations:** 1 BC Rotary Hearing and Balance Centre at St. Paul’s Hospital, University of British Columbia, Vancouver, British Columbia Canada; 2 Division of Otolaryngology-Head and Neck Surgery, BC Rotary Hearing and Balance Centre, St. Paul’s Hospital, 1081 Burrard St, Vancouver, B.C V6Z 1Y6 Canada

**Keywords:** Aerosol, COVID-19, PPE, PAPR, Respirator, Guideline, Pandemic, Review

## Abstract

**Background:**

Severe acute respiratory syndrome coronavirus 2 (SARS-CoV-2), the virus responsible for Coronavirus disease 2019 (COVID-19) has a predilection for infecting the mucosa of the upper and lower airways. Otolaryngologists and supporting health care workers (HCWs) are particularly at high risk of becoming infected while treating patients as many in-office procedures and surgeries are Aerosol Generating Medical Procedures (AGMP). Based on a review of the literature and various guidelines, recommendations are made to mitigate the risk to health care workers of becoming infected with SARS-CoV-2 while providing clinical care.

**Recommendations:**

During the COVID-19 pandemic all elective and non-time sensitive Otolaryngology procedures should be deferred to mitigate the risk of transmission of infection to HCWs. For non-AGMPs in all patients, even COVID-19 positive patients Level 1 PPE (surgical mask, gown, gloves and face shield or goggles) is sufficient. If local prevalence is favourable and patients are asymptomatic and test negative for SARS-CoV-2, Level 1 PPE can be used during short duration AGMPs, with limited risk of infected aerosol spread. For AGMPs in patients who test positive for SARS-CoV-2 a minimum of Level 2 PPE, with adequate protection of mucosal surfaces, is recommended (N95/FFP2 respirator, gown, double gloves, goggles or face shield and head cover). For long duration AGMPs that are deemed high-risk in COVID-19 positive patients, Level 3 PPE can provide a higher level of protection and be more comfortable during long duration surgeries if surgical hoods or PAPRs are used. It is recommended that these procedures are performed in negative pressure rooms, if available. It is essential to follow strict donning and doffing protocols to minimize the risk of contamination.

**Conclusions:**

By following strict infection prevention recommendations, the risk of HCWs becoming infected with SARS-CoV-2 while treating patients can be minimized. As the COVID-19 pandemic evolves rapidly, these recommendations should serve as guidance and need to be interpreted based on local factors and availability of healthcare resources.

## Background

Severe acute respiratory syndrome coronavirus 2 (SARS-CoV-2), the virus responsible for Coronavirus disease 2019 (COVID-19) has a predilection for infecting the mucosa of the upper and lower airways [1–4]. Therefore, the viral load is very high in the mucosa of the nasal cavities, pharynx and oral cavity, and (presumably) the Eustachian tube and middle ear/mastoid mucosa [1, 2].

Airborne transmission occurs during transmission of small, inspirable aerosols (< 5 um) containing infectious pathogens [5–7]. Once airborne, these aerosols can remain infective over time, can spread over extensive distances by air currents and may eventually infect susceptible persons who have not had face-to-face contact with the infectious individual [5–7]. Infectious pathogens which can spread by airborne transmission include *Mycobacterium tuberculosis* and rubella virus (measles) [5–7]. Droplet transmission refers to transmission of infection by (larger) aerosols over short distances directly from the respiratory tract of an infected person to mucosal surfaces of the susceptible person [5–7]. Examples of infectious agents primarily transmitted by the droplet transmission include influenza virus, rhinovirus and SARS-CoV-1 [5, 7, 8]. The distinction between droplet and airborne transmission is not precise and there are concerns about potential short-distance transmission by small, inspirable aerosols present in the respiratory cloud [5, 9].

Although SARS-CoV-2 is primarily droplet spread, many procedures on the upper airway are at high risk of generating aerosolized particles [10]. Once aerosolized, SARS-CoV-2 may remain viable in the air for at least three hours, implying potential airborne transmission [11]. However, the clinical relevance of this study’s results have been questioned given the methods used to aerosolize the particles and clinical evidence demonstrating SARS-CoV-2 infection from airborne transmission is lacking.

Since the diameter of SARS-CoV-2 is only 60–140 nm it can easily be transported in aerosols of varying size [12]. When working in close proximity to the infected patient, both larger droplets and small inspirable aerosols can impose a significant risk. When performing Aerosol Generating Medical Procedures (AGMP) the contamination risk is even higher [4, 13–16]. Anecdotal reports from China and Iran, suggest that Otolaryngologists and other health care workers (HCWs) around them when performing AGMPs are most frequently exposed and infected by SARS-CoV-2 [17]. It is therefore essential that Otolaryngologists are aware that many in-office procedures and surgeries, are AGMPs, including:

- Endoscopic examinations and sinonasal procedures. Nasopharyngoscopy, laryngoscopy and bronchoscopy are high-risk AGMP, especially given high SARS-CoV-2 viral loads in the upper and lower respiratory tracts in infected patients [18, 19]. During endoscopic sinus and skull base surgeries, frequent saline irrigations and the use of microdebriders can create aerosolized particles. Additionally, control of epistaxis can be associated with sneezing and coughing by the patient with the health care provider in very close proximity.
- Mastoid surgery. Drilling of the mastoid bone results in significant aerosolization that cannot be contained [20]. Various viruses, including coronaviruses, have been documented in the middle ear mucosa during active infections [21, 22].
- Head and neck mucosal cancer surgery should be considered as AGMP given the high viral load in the pharynx. Non-mucosal cancer surgery (salivary gland, thyroid) probably does not carry the same risk.

In the early phases of epidemics and pandemics with droplet or airborne transmitted pathogens, all elective and non-time sensitive Otolaryngology procedures should be deferred to minimize use of health care resources as well as to minimize risk of spread of infection to HCWs during any procedures [23]. However, not all medical procedures can be deferred. When needed to be performed, HCWs need to be aware of the risk of infection to themselves and to others, and more importantly, how to mitigate that risk through the proper use of personal protective equipment (PPE). While minimizing the risk of transmission to HCWs, it is also incumbent on the HCW to preserve scant heath care resources during a pandemic as availability of PPE may be finite.

The objective of this paper was to review the literature, including the different guidelines and recommendations to provide a much-needed framework to protect health care workers from the spread of SARS-CoV-2 during procedures on the upper airway during the COVID-19 pandemic.

### Recommendations for minimum PPE for health care workers during COVID-19 pandemic

The recommendations for minimum PPE for otolaryngology HCWs performing non-AGMP and AGMP procedures on patients during and after the COVID-19 pandemic are summarized in Tables 1 and 2. These recommendations should serve as guidance and need to be interpreted based on local factors: prevalence of COVID-19 in the community, if known; availability and timeliness of SARS-CoV-2 testing; accuracy of SARS-CoV-2 test used locally; and the availability of healthcare resources, including PPE, negative pressure rooms and HCWs. It will be important for HCWs to work closely with their Infection Prevention and Control experts to determine what is most appropriate for their local situation. Standard precautions, including hand hygiene, cleaning and disinfection of equipment and environment are essential during this outbreak response [10]. Increased awareness of potential surface and contact spread can improve compliance of HCWs for standard precautions, mitigating the risk of transmission.

**Table 1:** Summary of recommendations for minimum Personal Protective Equipment for Health Care Workers during COVID-19 Pandemic

	Level 1 PPE	Level 2 PPE	Level 3 PPE
**FOR NON-AGMP PROCEDURES**			
Asymptomatic + SARS-CoV-2 negative or unknown	**X**		
Symptomatic + SARS-CoV-2 negative	**X**		
Symptomatic + SARS-CoV-2 positive or high risk	**X**^**a**^	**X**^**a**^	
**FOR AGMP PROCEDURES**			
Asymptomatic + SARS-CoV-2 negative	**X**^**b**^	**X**^**b**^	
Asymptomatic + SARS-CoV-2 pending		**X**	
Symptomatic + SARS-CoV-2 negative		**X**	
Symptomatic + SARS-CoV-2 pending due to urgency		**X**^**c**^	**X**^**c**^
Symptomatic + SARS-CoV-2 positive		**X**^**c**^	**X**^**c**^

^a^This depends on the type and duration of examination and procedure. When there is a prolonged exposure of the HCW within the respiratory cloud of the patient, Level 2 PPE is advised

^b^This depends on the local COVID-19 prevalence, test reliability and type and duration of AGMP. In favourable situations Level 1 PPE is adequate

^c^This depends on the type and duration of AGMP and patient population: for a short duration, low risk AGMP, like intubation, Level 2 PPE is adequate, whereas for procedures with prolonged aerosol formation, like sinonasal surgery using drills, a higher level of protection may be warranted

**Table 2:** Summary of Personal Protective Equipment levels for Health Care Workers during COVID-19 Pandemic

Level 1 PPE	Level 2 PPE	Level 3 PPE
**Surgical mask**	**N95/FFP2**	**PAPR or N95/FFP2 + surgical mask**
**Gown**^**b**^	**Water impermeable gown**	**Gowns:** **1. coverall +gown**^**a**^ **2. water impermeable gown**
**Gloves**	**Double gloves**	**Double gloves**
**Face shield / goggles**^**b**^	**Goggles / face shield**	**Goggles + (face shield)**
**Head cover (optional)**	**Head cover, including neck protection**	**Head cover, including neck protection**

^a^Coverall with integrated hood and boots is preferred over gown with separate boot and leg covers and head-neck cover, since it reduces the risk of self-contamination during doffing and will provide optimal protection. A single layer surgical water impermeable gown (AAMI level 4), with a surgical hood or PAPR, and separate boot and leg covers, will provide a similar level of protection. If surgical hoods and PAPRs are not available or cannot be used during the procedure, a surgical gown (AAMI level 4) with properly fitting head and neck cover and goggles will provide adequate protection. Coveralls have to be used in conjunction with a second sterile surgical gown, when used during surgery

^b^Gown and face shield/goggles are recommended when providing direct patient care to COVID-19 patients and optional for in office non-AGMPs in negative or low risk patients, and only advised if there is a risk of fluid spread

In addition, one should be aware that a single negative test does not mean the patient is not infected. Two tests separated by 24 h pre-operatively, are recommended by some given a suggested sensitivity of nasopharyngeal swab testing of 70% [24, 25]; the sensitivity of the test is dependent on the adequacy of the sample obtained during nasopharyngeal swabbing as well as the technical sensitivity of the test itself with different tests having different sensitivities. As an example, if a test has a sensitivity of 0.70 (and assuming a specificity of 1.0 or 100%) and pre-test SARS-CoV-2 infection is present in 10% of the population, then the post-test likelihood the patient is infected with SARS-CoV-2 after a negative test is 3%. During a pandemic, there may be limited resources available for testing, precluding even a single test being performed.

### Patients who are asymptomatic and SARS-CoV-2 negative or unknown (low risk)

During the COVID-19 pandemic, Level 1 PPE should be used as a minimum for routine patient care and during non-AGMPs in all patients. Level 1 PPE includes a surgical mask, gown, gloves and eye protection (face shield or goggles). In addition, it is recommended that patients wear surgical masks and physical distancing should be maintained whenever possible. For asymptomatic and negative patients Level 1 PPE is advised. The use of a gown is optional in these patients, especially when there is no risk of droplet or fluid spread. The use of surgical masks, can interfere with essential communication in specific patient populations, like children or patients with severe hearing impairment. If the local COVID-19 prevalence is favourable and the patient is asymptomatic and/or test negative, an individual decision can be made by the HCW to (partially) discontinue the use of Level 1 PPE and when possible use physical distancing measures.

Ideally, all patients undergoing an AGMP including surgeries under general anesthesia should be screened for SARS-CoV-2; the act of endotracheal intubation (and extubation) is an AGMP. Even if they have no symptoms or risk factors for COVID-19, patients should undergo screening for SARS-CoV-2 within a few days prior to the procedure, after which time they are self-quarantined until the procedure is performed. Risk factors for SARS-CoV-2 infection have included recent travel history or contact with other persons known to be infected with SARS-CoV-2. However, with widespread community transmission now, travel history is no longer a defining risk factor.

Even if the patient tests negative, Level 2 PPE precautions should be followed for all AGMPs in situations with a high or growing COVID-19 prevalence. This is recommended given the possibility of false negative testing in a population with a high prevalence of COVID-19. Level 2 PPE includes the use of N95/FFP2 respirators, head cover including neck protection, eye protection preferably that seals to the face, double gloves and a water impermeable gown. If the local COVID-19 prevalence is favourable, local test reliability is good and the duration and/or risk of aerosol generation is low, then Level 1 PPE is adequate for asymptomatic and negative patients [10].

If patients cannot be tested either due to the urgency of the required medical procedure or due to the timeliness of available SARS-CoV-2 testing, then all asymptomatic patients undergoing AGMPs should be managed at a minimum using Level 2 PPE.

If N95 respirators are not available, the use of a surgical mask with goggles or face shield would be next best. A meta-analysis did not find a significant difference in preventing influenza respiratory viral infections when comparing surgical masks and N95 respirators [26]. However, SARS-CoV-2 in many ways behaves differently from influenzae; as the reproduction number and severity of COVID-19 is higher than seen with seasonal influenza [27–29], (and AGMPs differ in method of transmission) the results of this meta-analysis might not be applicable to COVID-19 and must be interpreted with caution.

### Patients who are symptomatic and SARS-CoV-2 negative (intermediate risk)

All procedures in symptomatic patients should be delayed, if possible, until testing for SARS-CoV-2 is performed and/or the patient’s symptoms have resolved. If another diagnostic procedure could be performed instead of an AGMP to achieve the desired results, then this should be done instead.

Given the possibility of false negative test results, HCWs performing non-AGMP should use a minimum of Level 1 PPE. However, if local resources allow it and COVID-19 prevalence is high, Level 2 PPE is preferable in symptomatic patients even if they test negative.

Given the possibility of false negative test results, we recommend that in situations with a high or growing COVID-19 prevalence, HCWs performing any AGMP use a minimum of Level 2 PPE in symptomatic patients even if they test negative. The use of Level 3 PPE should be considered in all these patients, and an individual risk assessment should be made including the patient’s symptoms, risk of aerosol formation, local prevalence of COVID-19 and local resources and protocols.

Appropriate protocols must be followed when donning and doffing PPE.

Additional preventative measures should also be considered: the number of people potentially exposed during the procedure should be limited to as few as possible; standard cleaning protocols should be strictly followed, including for instance immediate cleansing of instruments, flushing of any lumens of instruments to decrease the bioburden, immediate soaking in viricidal solutions (even soap and water seems effective for SARS-CoV-2), and appropriate cleansing of the room after the procedure according to local hospital protocols.

### Patients who are symptomatic and SARS-CoV-2 positive or unknown (high risk)

HCWs working in close proximity with SARS-CoV-2 positive patients should use a minimum of Level 2 PPE. If AGMPs are deemed urgent and cannot be delayed until the infection resolves, a minimum of Level 2 PPE is recommended. If available, the procedures should be performed in a negative pressure room, and the number of team members exposed should be minimized [14, 30].

Clinical experience indicates that in SARS-CoV-2 positive patients, Level 2 PPE provides adequate protection of HCWs during short duration AGMPs, such as endotracheal intubations [10, 35]. For long duration AGMPs that are deemed high-risk, like sinonasal procedures using drills, in SARS-CoV-2 positive patients, a higher level of protection is recommended, i.e. Level 3 PPE. Level 3 PPE provides even higher level of protection compared to Level 2 and is designed for procedures with long duration of aerosolization. Level 3 PPE consists of N95/FFP2 (or higher level respirator), goggles and a first layer of surgical gloves. This is followed by leg and foot covers, a long sleeved, sterile water impermeable gown (AAMI level 4) and sterile outer gloves. A surgical mask with attached face shield can be worn over the respirator to provide better protection of skin surfaces and limit contamination of the respirators. [14, 30–32]. If coveralls are used during surgery, then they have to be used in conjunction with a second sterile surgical gown. For long duration AGMPs, the use of PAPRs can provide an even higher level of protection and be more comfortable compared to respirators.

While donning and doffing PPE it is advised that a second HCW is present to assist and to ensure if the donning and doffing is performed correctly, as doffing can result in self-contamination even in experienced HCWs [14, 33]. Unfamiliarity of HCWs with these PPE poses a risk of self-contamination or virus spread during doffing. Donning and doffing training sessions are therefore essential parts of adequate infection prevention programs and should be coordinated with local Infection Prevention and Control experts. In addition, it is recommended that donning and doffing of PPE during the procedure should be minimized as much as possible to reduce contamination risk.

Contamination of mucous membranes is probably the most important mode of infection transmission. Hence, respirators and goggles are essential as they provide a tight seal minimizing the risk of direct aerosol transmission and can prevent accidental self-contamination caused by touching mucosal surfaces. Full face or hood Powered Air-Purifying Respirators (PAPRs) are designed to provide even higher protection against hazardous particles and to reduce the risk of potential face seal leakage, especially in those who cannot be successfully fit-tested with respirators [32, 34].

If respirators are used, then the use of N99/FFP3 respirators will provide a higher level of protection given their higher minimum filtration efficiencies (99%) to small (aerosolized) particles, compared to the N95/FFP2 respirators (94–95%). If N99/FFP3 respirators are not available an N95 mask seems adequate. Clinical experience during the first months of the COVID-19 outbreak, indicates that N95/FFP2 respirators provide adequate protection during AGMPs, like intubations [35].

Negative pressure rooms, or airborne infection isolation rooms (AIIR) are designed for isolation and treatment of patients with suspected or confirmed airborne infectious disease. The negative pressure prevents dissemination outside the room. Exhaust air from this room is filtered through HEPA filters, which are capable of filtering essentially all particles, including nanoparticles (< 0.01um) [5, 36]. It is important to work closely with the local Infection Prevention and Control experts to determine the appropriate use and logistics of available negative pressure rooms.

### Powered air-purifying respirators (PAPRs)

There is controversy regarding the clinical value of Powered Air-Purifying Respirators (PAPRs) or Controlled Air-Purifying Respirators (CAPRs) during aerosolizing procedures. PAPRs are designed to provide higher protection factors compared to N95 respirators and use HEPA filters (the minimum filtration efficiencies for 0.3um particles for N95/FFP2: 95% and HEPA: 99.97%) [34]. The APF provides an estimate of the level of protection for the various respirators and it includes both the filtration efficiencies of the used filters and potential face seal leakage [34]. According to the Occupational Safety and Health Administration (OSHA) standards, the Assigned Protection Factors (APF) for PAPRs range from 25 for loose fitting face-pieces to 1000 for full face-pieces, whereas the APF for N95 masks is 10 [34]. An APF of 10 means that one-tenth (10%) of contaminants to which the HCW is exposed can leak into the inside of the mask. For a PAPR with an APF of 50, this is only 2%. Although PAPRs have higher protection factors compared to N95 respirators in laboratory settings [37, 38], there is no clinical evidence indicating that PAPRs are more effective in reducing the risk of viral airborne transmission in clinical health care settings. Nevertheless, PAPRs can be used by HCWs who cannot be successfully fitted with N95 respirators, and can be more comfortable when worn for prolonged periods [31]. On the other hand there are several disadvantages to the use of PAPRs, as they are more expensive compared to respirators, impair communication due to the ventilator noise and tight seal, and require complex decontamination and maintenance procedures (Table 3). In addition, doffing of PAPRs can be more cumbersome and can result in contamination: a simulation study assessing the self-contamination risk during Ebola PPE doffing, revealed that even in experienced HCWs, improper removal of the PAPR, was one of the highest risk factors for self-contamination [33].

**Table 3:** Considerations regarding the use of Powered Air-Purifying Respirators (PAPRs). Adapted from Wax and Christian 2020

Potential advantages of PAPR	Potential disadvantages of PAPR
Higher protection factors and less breakthrough events (APF ≥ 25), compared to N95 respirators	Higher costs and limited availability
Do not require fit testing and can be used by HCWs who cannot be successfully fit tested with N95/N99 respirators or with facial hair	More difficult and time-consuming to don and doff, with a potential increased risk of self-contamination while doffing
Reusable, and can reduce the burden on respirator availability	Communication difficulties due to tight seal and ventilator noise
More comfortable for prolonged use	Requires complex decontamination procedures for reuse
Full facial and head cover (depending on model)	Depending on model, requires supply of disposable components, e.g. filters, hoses
	Can be difficult to use in combination with operating microscopes

## Discussion

To ensure the health and safety of HCWs during this COVID-19 pandemic we have proposed three levels for personal protection. For standard patient care and routine assessment of patients, during which no interventions are performed, even in patients known to be SARS-CoV-2 positive, Level 1 PPE (droplet precautions) is sufficient. Clinical experience indicates that Level 2 PPE (airborne precautions) provides adequate protection of HCWs during short duration AGMPs, such as endotracheal intubations, even if SARS-CoV-2 positive. For long duration AGMPs that are deemed high-risk, like sinonasal procedures using drills, in patients known to be SARS-CoV-2 positive, a higher level of protection is recommended, i.e. Level 3 PPE.

The emergence of COVID-19 presents challenges to provide adequate protection and prevent spread of infection among HCWs. Concerns regarding the potential routes of transmission, the severity of the disease and lack of effective treatments and vaccinations result in differing prevention strategies. In the early phases of a new pandemic with an unknown pathogen, extreme prevention strategies may be justified until (clinical) evidence reveals more details about transmission routes and the minimum set of precautions and PPE necessary to prevent transmission among HCWs.

After the SARS outbreak, Roy and Milton proposed an alternative classification of aerosol transmission of diseases [8]. *Obligate* airborne transmission is seen with M. tuberculosis, which is only transmitted through inhalation of small, inspirable aerosols. *Preferential* airborne transmission is seen with measles and varicella, in which multiple routes can lead to infection, but small aerosols are the predominant route. Finally, *opportunistic* airborne transmission is seen in diseases which are predominantly spread through other routes, but may be transmitted through small aerosols in rare occurrences as seen with influenza and SARS [8]. At the time of writing, there are two reports which identified SARS-CoV-2 RNA on air vents of patient wards [39, 40]. Although concerning for potential airborne spread of the virus the clinical relevance is uncertain; droplet precautions appear to be adequate for the protection of HCWs who provide standard patient care to COVID-19 patients and airborne precautions with N95/FFP2 respirators appear to provide sufficient protection during AGMPs [10, 35, 41]. These preliminary data suggest that SARS-CoV-2 may be classified as an *opportunistic* airborne transmitted pathogen: the predominant routes of transmission are through contact surfaces and aerosol spread over short distances, but occasional opportunistic airborne infections over long distances may occur. This would imply that airborne precautions with adequate protection of mucosal surfaces, is needed especially during procedures where aerosol formation is expected.

## Conclusion

During this COVID-19 pandemic, the health and safety of HCWs is essential to ensure ongoing care of patients and to prevent the collapse of health care systems. By following strict infection prevention recommendations, the risk of HCWs becoming infected with SARS-CoV-2 while treating patients can be minimized. As the regional and national situations change rapidly, these recommendations should serve as guidance and will need to be interpreted based on local factors, availability of healthcare resources and new data as they become available.

### Disclaimer

The original version of this document was approved by The Canadian Society of Otolaryngology - Head & Neck Surgery (CSO-HNS) as it was felt to be useful as guidance for its members. The information contained is based on information available at the time of writing (April 1, 2020). We recognize that the situation is evolving rapidly, so recommendations may change. The guidance included in this document does not replace regular standards of care, nor do they replace the application of clinical judgement to each individual presentation, nor variations due to jurisdiction or facility type. The CSO-HNS is not liable for the accuracy or completeness of the information in this document. The information in this document cannot replace professional advice.

## Data Availability

Data sharing is not applicable to this article as no datasets were generated or analysed during the current study.

## Abbreviations

CSO-HNS: Canadian Society of Otolaryngology - Head & Neck Surgery
HCWs: Health care workers
SARS-CoV-2: Severe acute respiratory syndrome coronavirus 2
COVID-19: Coronavirus disease 2019
AGMP: Aerosol Generating Medical Procedures
PPE: Personal protective equipment
FFP: Filtering facepiece
PAPR: Powered Air-Purifying Respirators
CAPR: Controlled Air-Purifying Respirators
HEPA: High-efficiency particulate absorbing
OSHA: Occupational Safety and Health Administration
RNA: Ribonucleic acid
AIIR: Airborne infection isolation rooms
